# Changes in the Drinking Water Microbiome: Effects of Water Treatments Along the Flow of Two Drinking Water Treatment Plants in a Urbanized Area, Milan (Italy)

**DOI:** 10.3389/fmicb.2018.02557

**Published:** 2018-10-31

**Authors:** Antonia Bruno, Anna Sandionigi, Marzia Bernasconi, Antonella Panio, Massimo Labra, Maurizio Casiraghi

**Affiliations:** ^1^ZooPlantLab, Department of Biotechnology and Biosciences, University of Milano-Bicocca, Milan, Italy; ^2^Metropolitana Milanese S.p.A., Milan, Italy; ^3^Bicocca cEnter of Science and Technology for FOOD, University of Milano-Bicocca, Milan, Italy

**Keywords:** drinking water microbiome, HTS, microbial ecology, biodiversity, environmental bacteria, groundwater

## Abstract

While safe and of high quality, drinking water can host an astounding biodiversity of microorganisms, dismantling the belief of its “biological simplicity.” During the very few years, we are witnessing an exponential growth in scientific publications, exploring the ecology hidden in drinking water treatment plants (DWTPs) and drinking water distribution system (DWDS). We focused on what happens to the microbial communities from source water (groundwater) throughout the main steps of the potabilization process of a DWTP, located in an urbanized area in Northern Italy. Samples were processed by a stringent water filtration to retain even the smallest environmental bacteria and then analyzed with High-Throughput DNA Sequencing (HTS) techniques. We showed that carbon filters harbored a microbial community seeding and shaping water microbiota downstream, introducing a significant variation on incoming (groundwater) microbial community. Chlorination did not instantly affect the altered microbiota. We were also able to correctly predict (through machine learning analysis) samples belonging to groundwater (overall accuracy was 0.71), but the assignation was not reliable with carbon filter samples, which were incorrectly predicted as chlorination samples. The presence and abundance of specific microorganisms allowed us to hypothesize their role as indicators. In particular, Candidatus Adlerbacteria (Parcubacteria), together with microorganisms belonging to *Alphaproteobacteria* and *Gammaproteobacteria*, characterized treated water, but not raw water. An exception, confirming our hypothesis, is given by the samples downstream the filters renewal, which had a composition resembling groundwater. Volatility analysis illustrated how carbon filters represented an ecosystem that is stable over time, probably bearing the environmental conditions that promote the survival and growth of this peculiar microbial community.

## Introduction

Drinking water treatment plants (DWTPs) assure high-quality drinking water through a combination of treatment processes aiming at the removal of chemical and microbiological contaminants. Nevertheless, DWTPs are a source of unexpected biodiversity, harboring complex microbial ecosystems, where species interact in multilevel networks. In general, these environmental microorganisms are widespread in the DWTP and in the distribution system and are difficult to characterize with a classical microbiological approach, that relies on cultured-based methods (Douterelo et al., 2014). Together with the advance in High-Throughput DNA Sequencing (HTS) analyses, we are now aware that the microbiota residing in the drinking water treatment and distribution system can have an impact on the biological quality of drinking water (Proctor and Hammes, 2015; Bautista-de los Santos et al., 2016; Zhang et al., 2017; Ling et al., 2018) and that bacterial community composition could have a link with the occurrences of opportunistic pathogens (Berry et al., 2006; Wang et al., 2013; Ji et al., 2017; Wang et al., 2017). In particular, drinking water potabilization processes can affect the native microbial community that naturally occurs in source waters (Pinto et al., 2012; Chao et al., 2013; Oh et al., 2018).

Despite this advances in the characterization of drinking water microbiota, the occurrence of uncharacterized environmental bacteria, some of which have been shown to have ultrasmall cell sizes (Brown et al., 2015; Luef et al., 2015), is common and underestimated in many environments, including DWTPs. One of the reasons is procedural: most analyses start from water filtration that allows the retaining of >0.2 μm microorganisms only.

Considering for example groundwater as source water, only recently we could appreciate the astonishing biodiversity of microorganisms, most of them belonging to candidate phyla and some of them with very small dimensions (less than 0.2 μm) and reduced genomes (around 1 Mb). This incoherent group of prokaryotic organisms is not marginal, probably comprising more than 15% of the whole Bacteria domain (Brown et al., 2015).

The role and the impact of potabilization process on such microorganisms inhabiting groundwater ecosystem and DWTP has not yet been fully elucidated. Thus, we focused our attention on the microbial community structure changes, starting from untreated groundwater and after the main potabilization processes of two DWTPs (located in a urbanized area of Northern Italy): granular activated carbon filter for the removal of chemical contaminants and the final disinfection step mediated by chlorination.

Interestingly, in the last years some authors tried to describe treatment and distribution processes as ecological disturbances, exhibited over space on the microbiome continuum in a groundwater–derived system (Zhang et al., 2017). Starting from the seminal paper of Pinto et al. (2012), where the fundamental role of granular activated carbon filter in shaping the downstream microbial community inside the DWTP was established, we now know that carbon filters harbor a microbial community that differs from the upstream community (Gibert et al., 2013; Bruno et al., 2017), and some studies revealed the predominance of *Bradyrhizobiaceae* family and the enrichment in bacteria carrying functions associated with aromatics degradation, many of which were encoded by *Rhizobiales* (Oh et al., 2018). Considering the final disinfection step, it is well established that different compounds exhibited different degrees of effectiveness in shaping the microbial composition through chemically oxidizing bacterial cells and suppressing bacterial growth (Gagnon et al., 2004; Gomez-Alvarez et al., 2012; Hwang et al., 2012).

In general, bacterial growth is positively influenced by higher water temperatures, lower chlorine residuals, and less nutrient (carbon, phosphorus, nitrogen, and iron) limitation, while this is significantly different between samples of different origin (groundwater vs. surface water) (Nescerecka et al., 2018). Nevertheless, a core bacterial community was observed in water samples collected along the drinking water distribution system, independently of the characteristics of the incoming water and differences in hydraulic conditions between sites and over time, suggesting that internal factors are central in shaping biofilm formation and composition (Douterelo et al., 2017).

Finally, few studies encompassed long-term sampling campaign to evaluate if temporal trends are exhibited by the microbial community structure in drinking water (Pinto et al., 2014; Potgieter et al., 2018).

Given these premises, we conducted a 1-year sampling campaigns, aimed to move toward the investigation of drinking water microbiome by a stringent water filtration (more stringent than the traditional 0.2 μm pore size filtration) to recover even the smallest environmental microorganisms, coupled with HTS techniques.

We explored, in a previous study (Bruno et al., 2017), the neglected biodiversity of water microbiome inside DWTP, focusing only on the so-called “microbial dark matter” (adopting the definition of Solden et al. (2016). Here, we present the analysis of the overall microbial community, aiming at discovering if also the “bright side” of the microbial community residing in the DWTP covers the same pivotal role and contributes in describing the dynamics of this peculiar ecosystem.

## Materials and Methods

### Study Site

We considered for our analyses two DWTPs (Site 1 and Site 2) located in Milan, a urbanized area in Northern Italy.

Milan lies on an alluvial plain, where both agricultural and industrial activities are widespread.

The groundwater area of Milan is about 2000 km^2^, and it is the main source of drinking water for the metropolitan area. The subsoil is characterized by Pliocene-Pleistocene sediments of fluvial-glacial origin. Aboveground, the main lithotypes are constituted by sands and gravels. Going deeper, grain sizes lower, and permeability decreases. These sediments reach about 100 m of depth and constitute the so-called “Traditional Aquifer,” exploited by municipal water supplies with the majority of captation wells. We can distinguish three stratigraphic units: the deepest (the third aquifer) is about 100 m of depth, it is characterized by low or intermediate permeability sediments (silt and clay, with fractions of sand) and it is confined. The captation wells of the DWTPs we selected draw water from the third aquifer (Masetti et al., 2007).

The main potabilization steps of the DWTPs studied involved (i) the use of granular activated carbon filters for chemical contaminants removal through adsorption and (ii) disinfection through the addiction of chlorine.

Several environmental variables were measured (e g., weather, external temperature, and humidity) and are reported in Supplementary Table S1.

### Water Sampling

We conducted a year-long sampling campaign (from December 2013 to November 2014), collecting monthly water samples from Site 1. We also collected samples from a second DWTP (Site 2) in October–November 2014 (the same sampling days of Site 1).

Water samples belonged to different steps of the potabilization processes: (i) from groundwater (Aquifer), (ii) after the passage through the granular activated carbon filters (CFilters), and (iii) after chlorination (Chlor).

Considering the length of the sampling campaign of Site 1, the granular activated carbon filters encompass a level of usage from intermediate to exhausted (October 2014). One event that occurred during the sampling campaign is noteworthy: in Site 1 DWTP, granular activated carbon filters were renewed after October 2014, leading to new carbon filters for the sampling of November 2014. Site 2 was characterized by an intermediated level of usage of granular activated carbon filters.

We generated 42 samples, listed in Supplementary Table S1. All samples were aseptically collected in sterile bottles and kept cool and dark during transport to the laboratory. Chlorinated samples were collected using sterile bottles containing sodium thiosulfate (Na_2_S_2_O_3_, 20 mg/L), a dechlorinating agent.

Chemical, physical, and microbiological tests were conducted by the drinking water company MM S.p.A. (Supplementary Tables S2–S5), according to the Italian law D. Lgs. n. 31 of 2 February 2001 (implementing the European Directive 98/83/CEE).

### Sample Concentration and DNA Extraction

Samples were processed following the protocol tested in Bruno et al. (2016). Briefly, in order to reduce the volume of the samples and therefore concentrate the bacteria, we used a tangential flow filtration (TFF) system. The system involves a peristaltic pump (Masterflex L/S Economy Drive), Tygon^®^ tubing, sterile reservoirs and filtration modules. The tangential flow filter used was a VivaFlow 200 cassette (Sartorius) composed of polyethersulfone (PES) with a nominal pore rating of 10000 MWCO (Molecular Weight Cut Off) and a surface area of 200 cm^2^. We selected this nominal pore rating for the stringent filtration conditions required to collect even the smallest environmental bacteria (10000 MWCO allows the retention of particles <0.1 μm in size). The system was scaled up with an additional unit connected in parallel to increase the filtration surface area and the flow speed.

All tubing, tubing connections and containers were sterilized with sodium hypochlorite or autoclaved prior to each experiment. Every step was conducted in the laminar flow cabinet.

The TFF system was run at a transmembrane pressure of 1.5 bar. TFF experiments were carried out within 24 h after sampling, and samples were always kept at 4°C. For each sampling point, seven liters of water were concentrated to obtain 100 mL of operative volume.

Three aliquots of filtrate (that should not contain bacteria) were conserved for further tests to exclude the presence of bacteria.

DNA extraction was carried out for all the samples, including filtrate samples and extraction negative controls, with an automated instrument (NucliSens^®^ EasyMAG^TM^ system, Biomerieux Italia S.p.A., Florence, Italy), based on magnetic beads. Starting from 1 mL of sample, the nucleic acids were eluted in a final volume of 50 μL of elution buffer and stored at -80°C.

### qPCR Amplification of 16S rRNA Gene

Quantitative Real Time PCR (qPCR) assays were performed with AB 7500 (Applied Biosystem) targeting the same 16S rDNA region chosen for HTS, as described in Bruno et al. (2016). All water samples, samples deriving from filtrate of TFF (that should not contain cells) and DNA extraction negative controls were tested.

Briefly, qPCR conditions included an initial denaturation at 95°C for 10 min, followed by 40 cycles of denaturation at 95°C for 15 s and annealing-elongation at 55°C for 1 min. A final dissociation stage was performed. Amplification reaction consisted of 5.0 μl SsoFast EvaGreen Supermix with Low ROX (Bio-Rad S.r.l., Segrate, Milan, Italy), 0.1 μl each 10 μmol l^-1^ primer solution, 2 μl DNA sample, and 2.8 μl of Milli-Q water. All samples and negative controls (no template) were run in triplicate.

Ct (Threshold Cycles) values were converted in counts (DNA copies) using the formula:

Count=E(CtI−Ct)

Where E is the efficiency of amplification (data from Bruno, 2016) and Ct1 is the number of qPCR cycles required to detect a single target molecule. A one-way analysis of variance ANOVA in combination with Tukey *post hoc* tests was used to find significant differences among sampling points in bacterial DNA concentration. A probability of *P* < 0.05 was considered to indicate a significant difference.

### Library Preparation and Sequencing

Illumina MiSeq 16S (V3-V4 region of 16S rRNA gene) libraries were generated following standard protocol (16S Metagenomic Sequencing Library Preparation, Part # 15044223 Rev. B) with modifications described in Bruno et al. (2017), due to the low DNA concentrations. DNA extracts were normalized on Ct values of Real Time PCR with the same primer pairs, instead of measuring the total amount of microbial DNA with fluorometric/spectrophotometric methods. Negative controls were included in library preparation.

Samples were sequenced using the 2 × 300 paired-end chemistry (MiSeq Reagent Kit v3). Technical replicates were included to verify the sequencing reproducibility (84 samples in total). The sequencing process was conducted by National Research Council, Institute of Biomedical Technologies (CNR-ITB, Italy).

### Microbial Composition and Community Structure Analysis

The raw paired-end FASTQ reads were imported into the Quantitative Insights Into Microbial Ecology 2 program (QIIME2, ver. 2017.9.01^1^; Caporaso et al., 2010) and demultiplexed native plugin. Raw reads were subsequently deposited into the National Center for Biotechnology Information (NCBI) Sequence Read Archive (SRA) database (see Data Availability paragraph). The Divisive Amplicon Denoising Algorithm 2 (DADA2) (Callahan et al., 2016) was used to quality filter, trim, denoise, and mergepairs the data. Chimeric sequences were removed using the consensus method. The taxonomic assignment of the representative sequences, to obtain the Operational Taxonomic Units (OTUs), was carried out using the feature-classifier^2^ plugin implemented in QIIME2 against the SILVA SSU non-redundant database (132 release), adopting a consensus confidence threshold of 0.8.

Multibar plots were generated with the QIIME2 dedicated plugin taxa^3^.

Community analyses (beta diversity) were performed with quantitative (weighted UniFrac; Lozupone et al., 2007) distance metrics (evenly sampled at 8,000 reads per sample) using the *diversity* QIIME2 plugin. Statistical significance among groups (sampling site and treatment plant) was determined by the ADONIS (permutation-based ANOVA, PerMANOVA) test (Anderson, 2005) with 999 permutation-based weighted UniFrac distance metrics. PerMANOVA Pairwise contrast was performed by the beta-group-significance command of *diversity* plugin. We decided to adopt an ordination approach to explore the structure of microbial communities and specifically, we used principal coordinates plots (PCoA). The representative sequences were aligned with MAFFT and used for phylogenetic reconstruction in FastTree (Price et al., 2010).

The Random Forest classifier implemented in the *sample-classifier* QIIME2 plugin^4^ was used to predict a categorical sample metadata category (i.e., sampling point). The number of trees to grow for estimation was set to 1,000. Overall accuracy (i.e., the fraction of times that the tested samples are assigned the correct class), was calculated for each factor. K-fold cross-validation was performed during automatic feature selection and parameter optimization steps. A fivefold cross-validation was also performed. The feature table used to train the classifier was collapsed to genus level. Heatmap visualization was used to explore genera chosen for sample prediction in the supervised learning method.

We used volatility analysis (Bokulich et al., 2017) to examine how variance in Shannon diversity and Sampling Point category changes across time. This allowed us to assess how volatile a dependent variable is over a continuous, independent variable (e.g., time) in one or more groups. Native QIIME2 plugin^5^ was used to plot an interactive control chart.

## Results

### Water Parameters

The main chemical, physical and microbial analyses conducted by MM S.p.A. are reported in Supplementary Table S2. In particular, the mean water temperature of groundwater was 15°C, the free chlorine concentration in chlorination samples was on average 0.05 mg/L and pH values ranged from 7.4 and 8.

### Bacteria Quantification

**FIGURE 1:**
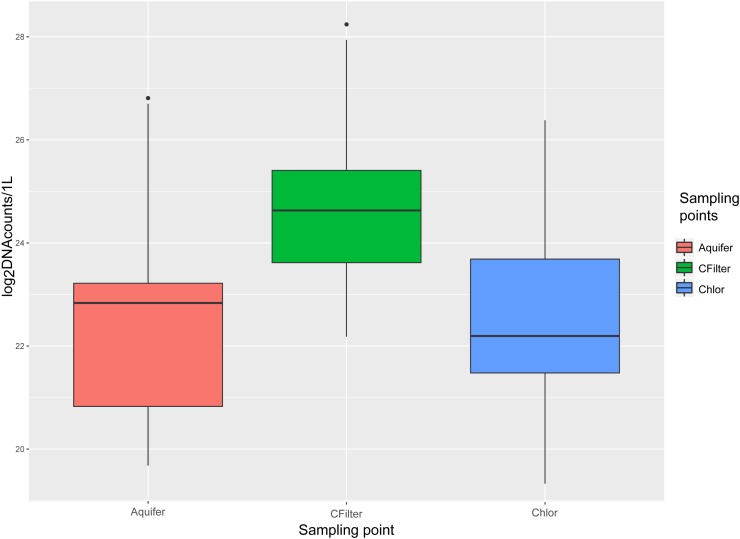
16S rDNA quantification for sampling points of Site 1. Values are expressed as log_2_(average of DNA counts)/L. Aquifer: groundwater samples; Cfilters: samples collected after the passage through granular activated carbon filters; Chlor: post-chlorination samples.

Bacteria were quantified for each sample through qPCR amplification of V3-V4 regions of 16 rDNA gene. DNA copies/L are reported in (Figure 1) and Supplementary Table S6. Melting temperatures (Tm) measured after the dissociation stage varied from 83.8–85.9°C.

Carbon filters sampling points significantly differed from groundwater and chlorination sampling points (*P* < 0.01), whereas there was not a significant difference between groundwater and chlorination sampling points (Supplementary Data S1 and Supplementary Figure S1). Statistical analyses demonstrated the significant difference (ANOVA, Tukey *post hoc* test: *P* < 0.01) among the three sampling points, for all the months tested, except for January, November, and December, when post-chlorination samples were not significantly different from groundwater samples. In July, post-chlorination samples were not significantly different from carbon filters samples and showed a 1.4-fold increase in 16S rDNA gene copies in respect of groundwater samples. Considering only Site 2, statistical analyses demonstrated the significant difference (ANOVA, Tukey *post hoc* test: *P* < 0.01) among the three sampling points, for all the months tested. In October 14, Site 2, groundwater bacterial concentration was higher than carbon filters and chlorination bacterial concentration. This behavior was not recorded in November 14, Site 2. All the negative controls (no template) and filtrate from TFF (no cells) resulted in no amplification.

### Sequence Analysis

About 19 million reads (8,474,127 + 11,287,412) were obtained. After quality filtering, merging reads and chimera removal of the two Illumina runs, we got an average of 93,205 reads per sample with an average of the magnitudes of deviations of 76,415 reads. We obtained 10,261 ASVs (amplicon sequence variants Callahan et al., 2017). Negative controls for library sequencing were not included in the analysis since the very low amount of DNA copies.

### Microbiome Diversity and Distribution

A total of 46 bacterial phyla and 95 classes were identified (plus 5 Archaea phyla and 12 Archaea classes) (Supplementary Data S2).

Taxonomic analysis revealed that most of the sequences in all the samples were associated with the phyla *Proteobacteria* (43%) and Patescibacteria (41%), followed by uncultured Omnitrophicaeota (3%), *Chloroflexi* (1%), and *Cyanobacteria* (1%).

Looking inside the taxonomic level of Class, the most abundant were Parcubacteria with the 32% of sequences, *Gammaproteobacteria* (23%), *Alphaproteobacteria* (14%), *Deltaproteobacteria* (7%), and ABY1 (Patescibacteria) (5%).

Focusing on specific features, we observed that the most abundant feature was assigned to the Candidatus Adlerbacteria (Parcubacteria). Here, we adopted the generic term “feature,” which is more inclusive than the term OTU, intending any unit of observation of any data type (that can be an OTU, an amplicon sequence variant, etc.), according to the use of QIIME2 program. A microorganism belonging to the genus *Acidovorax* was recorded in all the samples, even in Site 2 samples, and it is also among the most abundant bacteria retrieved. It is also worth mentioning the presence in all the three sampling points of the phylum *Cyanobacteria*, and more specifically bacteria belonging to the group Melainabacteria, that lack of photosynthesis pathways (Di Rienzi et al., 2013; Soo et al., 2014). Site 2 groundwater samples are dominated by *Crenothrix*, a genus of methane oxidizing bacteria, belonging to the class *Gammaproteobacteria*.

**FIGURE 2:**
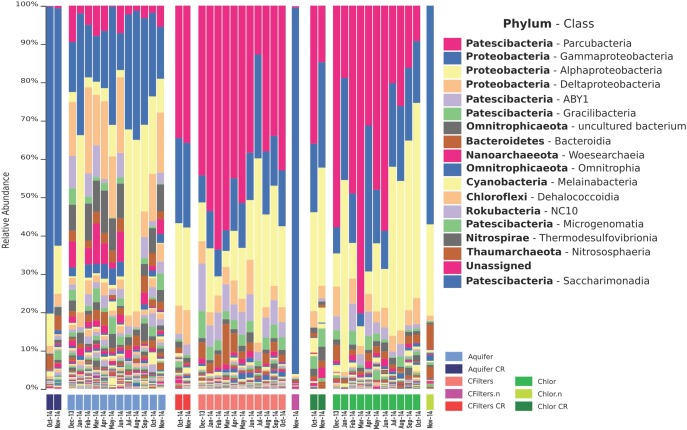
Barchart visualization depicting the relative abundance and distribution of the features assigned to class taxonomic rank Class. Aquifer: groundwater samples; Cfilters: samples collected after the passage through granular activated carbon filters; Cfilters.n: samples collected after the passage through renewed granular activated carbon filters; Chlor: post-chlorination samples; Chlor.n: post-chlorination samples after carbon filters renewal; CR: Site 2. The legend lists the 18 most abundant Classes.

Bar chart representation (Figure 2) highlights the distribution of Bacteria (and Archaea) classes, considering both Site 1 and Site 2. Groundwater samples are characterized by a high relative abundance of *Gammaproteobacteria*, *Alphaproteobacteria*, *Deltaproteobacteria*, and Parcubacteria. Carbon filters samples showed a switch in composition, with the predominance of Parcubacteria. This behavior was observed even in chlorination samples.

To better explore the microbial differences among sampling points, we computed beta diversity metrics and generated PCoA plots. To normalize the variance during the analysis, we set the even sampling depth to 8,000. The script that calculates beta diversity metrics uses this parameter to subsample the counts in each sample without replacement, so each sample in the resulting table has a total count of 8,000. If the total count for any sample is smaller than 8,000, the samples are excluded from the diversity analysis.

**FIGURE 3:**
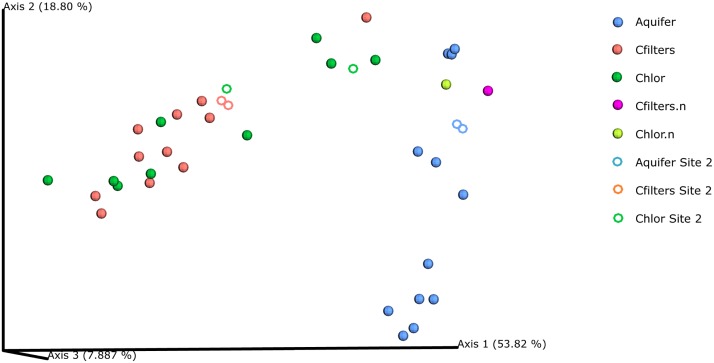
PCoA Emperor plots based on weighted UniFrac diversity metric. Water samples were compared based on sampling point. Aquifer: groundwater samples; Cfilters: samples collected after the passage through granular activated carbon filters; Cfilters.n: samples collected after the passage through renewed granular activated carbon filters; Chlor: post-chlorination samples; Chlor.n: post-chlorination samples after carbon filters renewal; Circles: Site 1; Rings: Site 2.

Weighted UniFrac-based PCoA plots (Figure 3) revealed a strong pattern of clustering of community structure by sampling point, depicting a clear separation between groundwater and carbon filters-chlorination microbiomes. Samples from groundwater clustered together, and within- sampling point UniFrac distances were generally smaller than between- sampling point distances, suggesting the community composition of samples from the same sampling point were more similar to each other. Moreover, samples belonging to carbon filters and chlorination samples clustered together and separately from groundwater samples. However, carbon filters and chlorination samples collected in November 14 (renewed carbon filters) plotted far distant from the other carbon filters and chlorination samples and adjacent to groundwater samples, confirming the evidences collected in the previous analyses (for sampling point: groundwater vs. carbon filters, pseudo-*F* = 19.4, *p* < 0.001; groundwater vs. chlorination, pseudo-*F* = 11.86, *p* < 0.001; and carbon filters vs. chlorination, pseudo-*F* = 0.82, *p* = 0.416).

### Machine Learning Analysis

**FIGURE 4:**
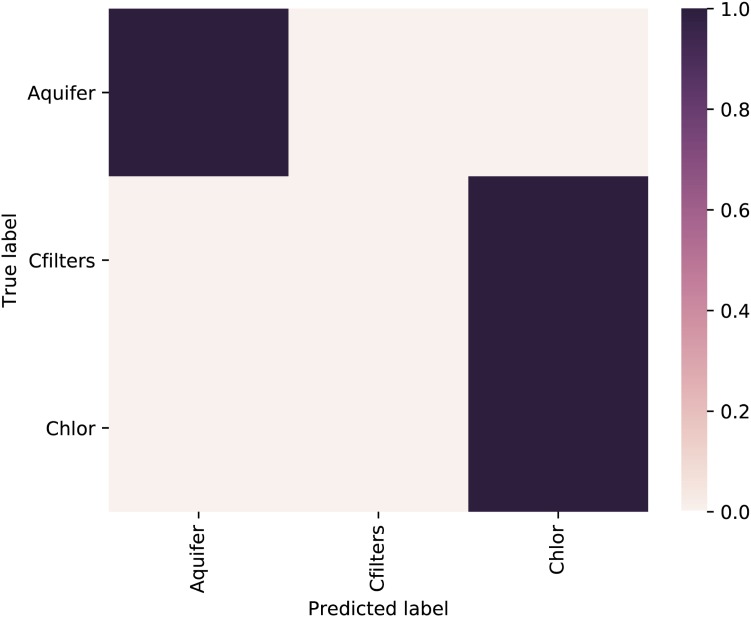
Heatmap of the confusion matrix showing the classification accuracy results of the supervised learning classifiers applied to sampling point metadata class. The feature table used to train the classifier was collapsed at the genus level. Aquifer: groundwater samples; Cfilters: samples collected after the passage through granular activated carbon filters; Chlor: post-chlorination samples.

A random forest was used as supervised learning classifier to predict sampling point category (Figure 4). We excluded from the analysis Site 2 samples and the samples collected after the event of carbon filter regeneration. Taxonomic diversity at the genus level was used as a trainer for the classifier. The comparison between “true label” vs. “predicted label” showed the highest probability to correctly predict the sampling point.

The overall accuracy was 0.714286 and the classifier correctly predicted the groundwater sampling points, but showed an incorrect assignation of carbon filters samples, which were predicted as chlorination samples.

The feature with the highest importance value (0.0575) for the model belongs to Candidatus Adlerbacteria (Parcubacteria) (Supplementary Data S3).

**FIGURE 5:**
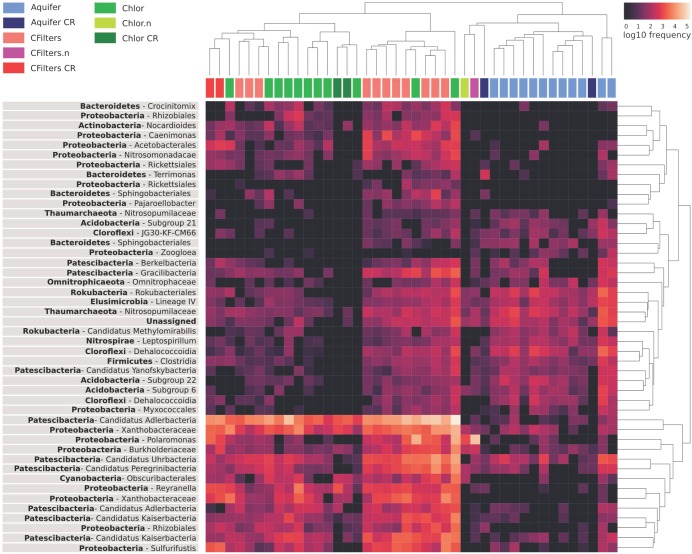
Heat map highlighting the relative abundance of the components of water microbiome mostly contributing to the correct prediction of sampling points in the machine learning analysis. Aquifer: groundwater samples; Cfilters: samples collected after the passage through granular activated carbon filters; Cfilters.n: samples collected after the passage through renewed granular activated carbon filters; Chlor: post-chlorination samples; Chlor.n: post-chlorination samples after carbon filters renewal; CR: Site 2.

To figure out which genera mostly contributed to distinguish the sampling points, we explored the distribution of the taxa that maximize the model accuracy of classifier with an heatmap (Figure 5). Analyzing the sample cluster dendrogram, two main clusters emerged.

The first one includes all samples belonging to groundwater, but also groundwater samples collected from a different DWTP (Site 2). Noteworthy, in the same cluster there are carbon filters and chlorination samples collected after the event of granular activated carbon filter renewal.

In the other cluster, carbon filters and chlorination samples are mixed even if they maintain a certain degree of structure.

Looking at the abundance (expressed as frequency) of each genus, the Candidate Adlerbacteria (Parcubacteria) showed high abundance in carbon filters-chlorination samples, but not in groundwater samples. Other microorganisms that exhibited a similar pattern are *Xhantobacteraceae*, *Polaromonas*, *Burkholderiaceae*, *Rhizobiales* (all *Proteobacteria*), and Patescibacteria candidate phyla.

### Volatility Analysis

The temporal stability or volatility of microbial communities among sampling points was measured, considering the whole duration sampling campaign, using Shannon diversity metric.

**FIGURE 6:**
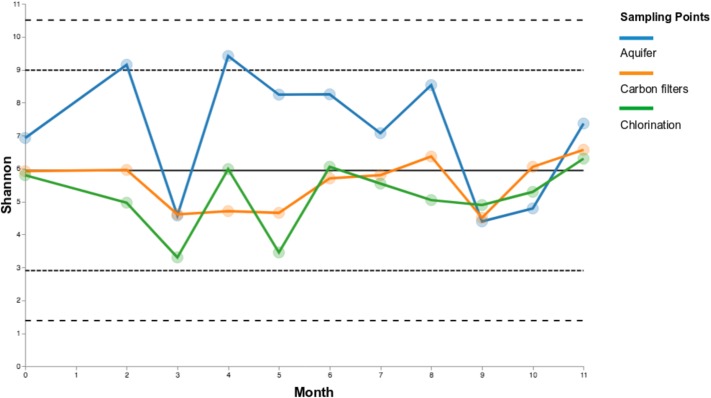
Volatility charts of Shannon diversity for sampling points over time (a) and the two groups “untreated water” (Treat_Y_N: no; groundwater) and “treated water” (Treat_Y_N: yes; carbon filters + chlorination) (b).

Carbon filters and chlorination samples exhibit similar degrees of variance in Shannon diversity, compared to groundwater samples, which show a less stability over time. This can be better appreciated looking at the groups of samples “untreated water” (Treat_Y_N: no), i.e., groundwater, and “treated water” (Treat_Y_N: yes), i.e., carbon filters and chlorination samples (Figure 6; in Supplementary Data S4 Shannon diversity is reported).

## Discussion

Understanding the microbial ecology of DWTPs is necessary to design innovative and effective control strategies that will ensure safe and high-quality drinking waters. Interactions between bacteria are unaccounted for in current disinfection models.

Drinking water emerging from the tap may contain bacteria (Pinto et al., 2012; Douterelo et al., 2014), archaea (van der Wielen et al., 2009), eukaryotes (Thomas and Ashbolt, 2010; Pereira et al., 2013; Zhang et al., 2017), and viruses (Lambertini et al., 2012), which together constitute a complex microbial community. Estimations indicate up to a few hundred millions of microbial cells per liter (Hammes et al., 2008).

### Bacterial Load Across the DWTP: A Preliminary Insight

From a quantitative point of view, water samples collected during our 1-year survey were all characterized by a very low concentration of microorganisms and this is consistent with previous studies involving groundwater (Douterelo et al., 2014; Luef et al., 2015).

From a methodological point of view, this represented a challenge, since quantification through absorbance-based methods (i.e., OD600 for intact cells or Nanodrop for DNA extracts) or fluorimetric methods (i.e., Qubit) was not reliable for the limit of sensitivity.

From a qualitative point of view, the composition of the microbial community revealed a high percentage of environmental bacteria, unculturable and often characterized by very small dimensions (see Bruno et al., 2017). Therefore, in this case study, quantification through plate count had strong biases and quantification through microscopy visualization showed low reproducibility (data not shown). Quantification based on 16S rRNA gene has some criticisms, related for instance to the presence of variable copy numbers in bacterial genomes (Větrovský and Baldrian, 2013) and to the impossibility to distinguish live from dead organisms. Nevertheless, we decided to use 16S-qPCR assay to quantify the bacterial load, to have a preliminary insight on quantitative shifts across the potabilization steps. qPCR data showed a significant increase in bacterial load after water passage through granular activated carbon filters. After chlorination the quantity of bacteria decreased, reaching values not significantly different from that of groundwater. Further analyses are needed to estimate the fraction of live microorganisms.

### Microbial Community Structure: High Diversity, Almost Unexplored

High-Throughput DNA Sequencing workflow enabled an accurate picture of the biological diversity present in water samples, starting from groundwater to drinking water. The advantages of HTS techniques are now well known, in particular when dealing with environmental bacteria, recalcitrant to growth on common media.

Taxonomic analysis of all the microbial world inside the two DWTPs investigated in this study revealed that most of the sequences in all the samples were associated with the phyla *Proteobacteria* and Patescibacteria, and a high degree of sequences of uncultured bacteria, still not characterized. The predominance of *Proteobacteria* is consistent with previous drinking water studies performed in different geographic locations (Pinto et al., 2012; Gomez-Alvarez et al., 2015; Oh et al., 2018) and using different techniques (Vandermaesen et al., 2017; Vaz-Moreira et al., 2017). Conversely, such a strong presence of Patescibacteria was not previously reported in studies focusing on drinking water microbiome.

From a microbiological perspective, the main objectives of drinking water treatments are to ensure the absence of any pathogenic bacteria in drinking water and to limit any uncontrolled regrowth during distribution of the water. It is important to underline that drinking water samples we collected were labeled as potable, according to the parameters provided by international directives (e.g., the European 98/83/CE) and safe for health. It is likely that Patescibacteria are not pathogenic, so why should an endeavor in studying these microorganisms be important?

One of the possible reasons of this effort is that the analysis of the forces that affect microbial dynamics provides new insights in the drinking water treatment process. It is therefore evident that a greater capacity of microbial organism identification is essential to address relevant improvement in prevention strategies. With our analysis, we discovered that groundwater harbors an astonishing biodiversity, that (1) is almost unknown and (2) significantly change after the passage through granular activated carbon filters and chlorination basin, (3) with no immediate effect of disinfection treatment on the community structure.

Considering the first result, a significant example of scarcity of knowledge regarding the drinking water microbiota is not only the presence of the candidate taxa belonging to Patescibacteria, but also the presence of the recently discovered Melainabacteria. We found features assigned to *Cyanobacteria* across the entire DWTP, an environment where photosynthesis does not occur. This could be surprising, except that recent studies (Di Rienzi et al., 2013; Soo et al., 2014) allowed the reconstruction of complete genomes for members of a new candidate phylum sibling to *Cyanobacteria*, called Melainabacteria, that are non-photosynthetic, anaerobic, motile, and obligately fermentative, with the capacity for nitrogen fixation using a nitrogenase distinct from that in *Cyanobacteria*. Organisms belonging to this phylum have been found in the human gut and in groundwater. Noteworthy, among the microorganisms that mostly contribute to the correct prediction of sampling points carbon filters-chlorination, we found in our analysis Obcuribacteriales, Melainabacteria representatives, that probably play a role in this peculiar ecosystem.

However, considering the second result, we demonstrated that native microbial communities deriving from groundwater are able to colonize carbon filters and significantly affect drinking water quality. Interestingly, similar evidences arose starting from different DWTPs, located in the same metropolitan area (as in our case study) or in different continents (as in Pinto et al., 2012), and through different DNA sequencing chemistries, supporting the robustness of results obtained. Our analyses demonstrated that Parcubacteria, and more specifically a Candidatus Adlerbacteria representative, significantly characterized treated water. Through machine learning analysis we correctly predicted samples belonging to groundwater or to treated water (carbon filters and chlorination treatment), giving an important insight into a predictive strategy to manage drinking water quality.

In the case of the treated water microbial community, we reported a certain level of stability along a temporal scale, in respect of the groundwater habitat, without measuring changes related to seasonality. Carbon filters represent an ecosystem that is stable over time, probably bearing the environmental conditions that promote the survival and growth of this peculiar microbial community.

Other recent studies (Pinto et al., 2014) showed opposite evidences: a seasonal pattern is exhibited by bacterial community and month and season were strong explanatory factors for changes in bacterial community structure.

In general, the bacterial taxa that we detected in our carbon filter samples are those sloughed off in the filter effluent and could be related to biofilm formation in carbon filters. Thus, the changes in the community structure associated with the carbon filters could be related to biofilm associated bacteria.

Previous studies (Lautenschlager et al., 2014) reported that the bacterial community structure in the filter effluent tends to resemble that in the filter. This tendency was observed only partially by Oh et al. (2018), suggesting that the discrimination of the filter bacterial communities from the effluent communities could be attributable to those particularly overrepresenting in filters. Understanding of the process of biofilm formation in carbon filters is not only fundamental for the delivery of safe water, but also an opportunity to control and manipulate filter microbiota and then modulate biofilm growth. Further investigations are needed to better describe the specific microbiota inhabiting carbon filter ecosystem and all the variables involved. In our specific case study, the possibility of Parcubacteria members to growth in biofilm or to have a role in biofilm formation should be investigated, since the significant predominance of this taxon in the carbon filter samples analyzed and the lack of knowledge about its metabolic capacities.

Finally, considering the third evidence, it is remarkable that the drinking water microbiome can persist under extreme conditions of chronic stress and very low substrate concentrations. To minimize detrimental effects caused by microbes, in DWTPs multiple hygienic barriers are employed, from ozonation, to UV disinfection, from inverse osmosis to chlorination. We can consider carbon filters as sources of chronic stress to water microbial community that probably select a peculiar microbiota, with specific functions. The chlorination process affected bacterial abundance, but not composition in the short term.

Due to the molecular techniques applied in our analysis, we did not distinguish live from dead bacteria and the implementation with flow cytometry or RNA-based assays will better elucidate the role of potabilization processes.

## Conclusion

Our data suggested that carbon filters are acting as a substrate enhancing microorganisms’ growth and contribute to seed water downstream, since chlorination do not modify greatly the incoming bacterial community in terms of global diversity. Pinto et al. (2012) and other researchers observed a similar pattern, but with different actors involved. We cannot exclude that this may derive from the use of membrane filters with a pore size ≥0.2 μm for the filtration process in those experiments and the consequent loss of a fraction of environmental microorganisms.

In general, the idea of applying machine learning analysis can be helpful not only in supporting the classic analysis of microbial diversity, but also (and more important) in trying to predict which taxa are most discriminating in response to a treatment. We verified the power of this analysis in predicting which samples belong to raw water (i.e., groundwater) or treated water (i.e., carbon filter and chlorination samples). The applications in the future, with a greater number of informations, could be several: the possibility of using microbial compositions to understand at which treatment step the sampled water belong (or which DWTP) or to see which taxa can be discriminating, leading to the identification of possible disturbing taxa or new microbial targets. Further analyses will aim at unraveling the complex network beyond drinking water microbial dynamics, elucidating the possibility of co-occurrence patterns: the microbiota residing across the DWTP and how it varies can be a not only a proxy of water quality, useful for monitoring by water companies, but also a prospective indicator, addressing prevention measures.

## Data Availability

Sequencing data were deposited in the National Center for Biotechnology Information (NCBI) Sequence Read Archive (SRA) under accession no. SAMN04364339-422.

## Author Contributions

AB, AS, MC, and ML conceived and designed the experiments and drafted the manuscript and figures. AB performed the experiments. AS analyzed the data. MB and AP contributed materials. All authors contributed to the revision of the final manuscript.

## Conflict of Interest Statement

The authors declare that the research was conducted in the absence of any commercial or financial relationships that could be construed as a potential conflict of interest.

## Footnotes

1 https://qiime2.org/

2 https://github.com/qiime2/q2-feature-classifier

3 https://github.com/qiime2/q2-taxa

4 https://github.com/qiime2/q2-sample-classifier

5 https://docs.qiime2.org/2018.4/plugins/available/longitudinal/volatility/
