# Supplementary material for: Changes in the Drinking Water Microbiome: Effects of Water Treatments Along the Flow of Two Drinking Water Treatment Plants in a Urbanized Area, Milan (Italy)
Source: Front Microbiol. 2018 Oct 31;9:2557. doi: 10.3389/fmicb.2018.02557 (PMC6220058; doi:10.3389/fmicb.2018.02557)
Supplement: Supplementary file 5 [file Data_Sheet_1.docx]

***Supplementary Material***

**Changes in the drinking water microbiome: effects of water treatments along the flow of two drinking water treatment plants in a urbanized area, Milan (Italy).**

**Antonia Bruno☨, Anna Sandionigi☨, Marzia Bernasconi, Antonella Panio, Massimo Labra, Maurizio Casiraghi***

^☨^ These authors contributed equally to this work.

*** Correspondence:** Corresponding Author: maurizio.casiraghi@unimib.it

# Supplementary Data

Supplementary Material should be uploaded separately on submission. Please include any supplementary data, figures and/or tables.

Supplementary material is not typeset so please ensure that all information is clearly presented, the appropriate caption is included in the file and not in the manuscript, and that the style conforms to the rest of the article.

# Supplementary Figures and Tables

**Supplementary Table S5**. Water samples microbiological analyses (data from MM S.p.A.).

| Sample_ID | Sampling Point Abbreviation | Coliforms at 37°C (MPN / 100 mL) | Enterococci (MPN / 100mL) | *Escherichia coli* (MPN / 100mL) | *Pseudomonas aeruginosa* (CFU / 250mL) | *Clostridium perfringens* (and spores) |
| --- | --- | --- | --- | --- | --- | --- |
| 3C | Chlor | 0 | 0 | 0 | 0 | NA |
| 4C | Chlor | 0 | 0 | 0 | NA | 0 |
| 5C | Chlor | 0 | 0 | 0 | 0 | NA |
| 6C | Chlor | 0 | 0 | 0 | NA | 0 |
| 7C | Chlor | 0 | 0 | 0 | 0 | NA |
| 8C | Chlor | 0 | 0 | 0 | NA | 0 |
| 9C | Chlor | 0 | 0 | 0 | 0 | NA |
| 10C | Chlor | 0 | 0 | 0 | NA | 0 |
| 11C | Chlor | 0 | 0 | 0 | 0 | NA |
| 12C | Chlor | 0 | 0 | 0 | NA | 0 |
| 13C | Chlor | 0 | 0 | 0 | 0 | NA |
| 14C | Chlor | 0 | 0 | 0 | NA | 0 |
| 1C_cr | Chlor | 0 | 0 | 0 | 0 | NA |
| 2C_cr | Chlor | 0 | 0 | 0 | NA | 0 |

The methods for microbiological parameters are:

(a) *Escherichia coli* and coliform bacteria (EN ISO 9308-1 or EN ISO 9308-2)

(b) Enterococci (EN ISO 7899-2)

(c) *Pseudomonas aeruginosa* (EN ISO 16266)

(d) *Clostridium perfringens* including spores (EN ISO 14189).

(a) The Colilert system (IDEXX Laboratories) was used according to the manufacturer’s instructions. 100 mL of drinking water was mixed with the substrate and incubated in a 51 well QuantiTray for 24 h at 37 ± 1°C. Total coliform-positive wells display a yellow colour, whereas *E. coli*-positive wells were yellow and fluoresce under UV light (365 nm). Counts of the number of positive wells were transferred to a Most Probable Number (MPN) of the target organisms.

(b) The Enterolert system (IDEXX Laboratories) was used according to the manufacturer’s instructions. 100 mL of drinking water was mixed with the substrate and incubated in a 51 well QuantiTray for 24 h at 41 ± 0.5°C. Total enterococci-positive wells fluoresce under UV light (365 nm). Counts of the number of positive wells were transferred to a Most Probable Number (MPN) of the target organisms.

(c) Membrane filtration followed by incubation of the membrane on CN agar at (36 ± 2) °C for (44 ± 4) h. Count all colonies that produce blue/green (pyocyanin) colour.

(d) Membrane filtration followed by anaerobic incubation of the membrane on m-CP agar at 44 ± 1 °C for 21 ± 3 hours. Count opaque yellow colonies that turn pink or red after exposure to ammonium hydroxide vapours for 20 to 30 seconds.

**Supplementary Table S6**. Bacteria quantification through qPCR. Mean values are reported, expressed as log_2_(DNAcounts)/L of water, and calculated as described in the main text; sd: standard deviation.

| **Sample_ID** | **Sampling Point Abbreviation** | **mean log2(DNAcounts)/1L** | **sd** |
| --- | --- | --- | --- |
| 3A | Aquifer | 22.83536 | 0.005774 |
| 3B | CFilter | 24.32536 | 0.456107 |
| 3C | Chlor | 23.19036 | 0.138564 |
| 4A | Aquifer | 22.52536 | 0.525389 |
| 4B | CFilter | 24.77536 | 0.433013 |
| 4C | Chlor | 22.96536 | 0.282902 |
| 5A | Aquifer | 26.75536 | 0.063509 |
| 5B | CFilter | 26.29036 | 0.023094 |
| 5C | Chlor | 26.16536 | 0.051962 |
| 6A | Aquifer | 21.96536 | 0.282902 |
| 6B | CFilter | 23.56536 | 0.121244 |
| 6C | Chlor | 24.43036 | 0.069282 |
| 7A | Aquifer | 23.34036 | 0.011547 |
| 7B | CFilter | 23.37036 | 0 |
| 7C | Chlor | 26.37536 | 0.005774 |
| 8A | Aquifer | 20.50536 | 0.271355 |
| 8B | CFilter | 24.31536 | 0.259808 |
| 8C | Chlor | 20.32536 | 0.017321 |
| 9A | Aquifer | 23.72536 | 0.075055 |
| 9B | CFilter | 25.34536 | 0.040415 |
| 9C | Chlor | 21.64036 | 0.034642 |
| 10A | Aquifer | 22.97036 | 0.046188 |
| 10B | CFilter | 23.45036 | 0.254034 |
| 10C | Chlor | 23.43036 | 0.034641 |
| 11A | Aquifer | 23.13536 | 0.051962 |
| 11B | CFilter | 25.52036 | 0.034641 |
| 11C | Chlor | 21.47536 | 0.005774 |
| 12A | Aquifer | 20.25036 | 0.658181 |
| 12B | CFilter | 25.18536 | 0.051962 |
| 12C | Chlor | 21.52036 | 0.023094 |
| 13A | Aquifer | 20.08036 | 0.381054 |
| 13B | CFilter | 22.23536 | 0.063509 |
| 13C | Chlor | 19.33036 | 0 |
| 14A | Aquifer | 20.87536 | 0.051961 |
| 14B | CFilter | 28.09036 | 0.173205 |
| 14C | Chlor | 21.19536 | 0.525389 |
| 1A Cr | Aquifer | 22.97036 | 0.046188 |
| 1B Cr | CFilter | 23.45036 | 0.254034 |
| 1C Cr | Chlor | 23.43036 | 0.034641 |
| 2A Cr | Aquifer | 23.13536 | 0.051962 |
| 2B Cr | CFilter | 25.52036 | 0.034641 |
| 2C Cr | Chlor | 21.47536 | 0.005774 |

**Supplementary Figure S1**

Bacteria quantification through qPCR. Mean values are reported, for each month, expressed as log_2_(DNAcounts)/L of concentrated water, and calculated as described in the main text.

**
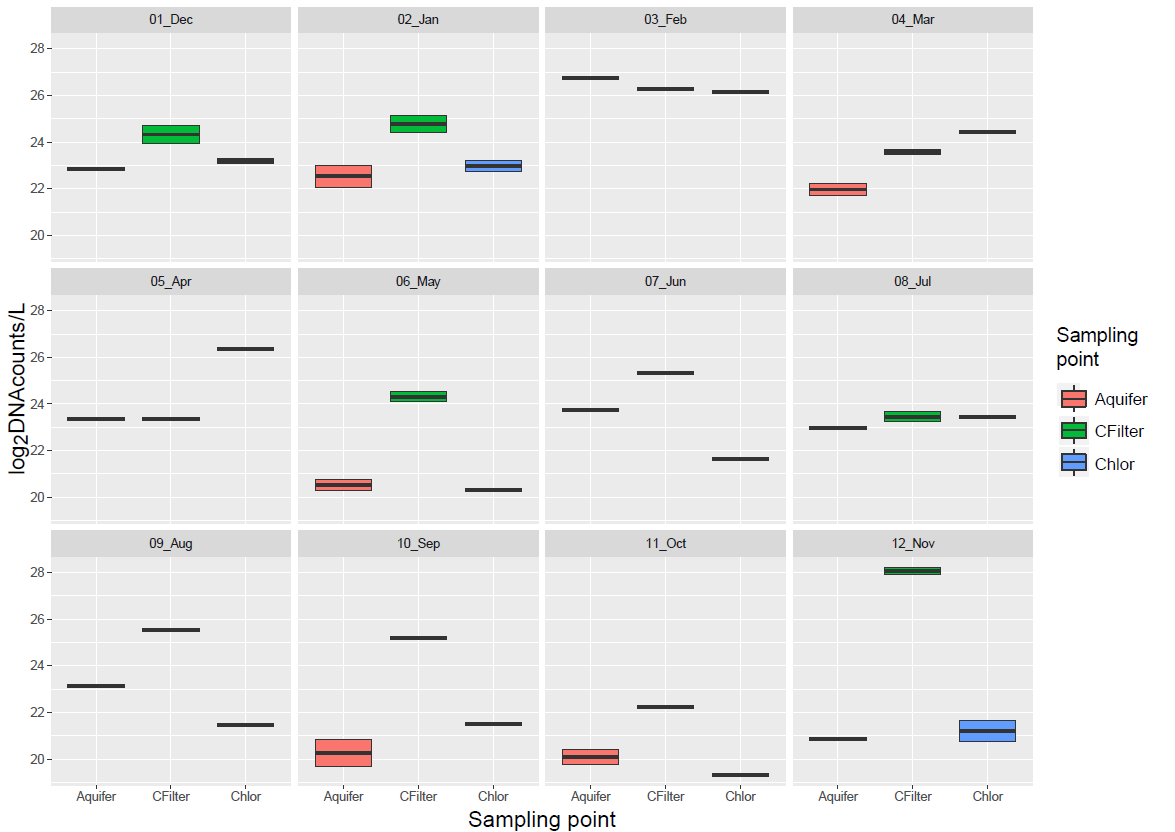
**

**Supplementary Data S1**

Statistical analysis for Sampling point, Site 1

ANOVA, Tukey post-hoc tests

Terms:

sampling_point Residuals

Sum of Squares 151.3991 476.9179

Deg. of Freedom 2 141

Residual standard error: 1.839129

Tukey multiple comparisons of means

95% family-wise confidence level

$sampling_point

diff lwr upr p adj

B.CFilter-A.Aquifer 2.2920836 1.4028395 3.181328 0.0000000 *

C.Chlor-A.Aquifer 0.2566669 -0.6325773 1.145911 0.7733769

C.Chlor-B.CFilter -2.0354167 -2.9246609 -1.146173 0.0000007*

***The following files are provided separately***

**Supplementary Table S1.**

Environmental variables recorded during the sampling campaign.

**Supplementary Table S2.**

Groundwater samples physico-chemical analyses.

**Supplementary Table S3.**

Carbon Filter water samples physico-chemical analyses.

**Supplementary Table S4.**

Post-chlorination water samples physico-chemical analyses.

**Supplementary_Data_S2.csv**

OTUs Assignment. For each unique OTU, (Feature ID), assigned Taxonomy are reported.

**Supplementary_Data_S3.csv**

Machine learning overall accuracy and significant discriminatory features (i.e., bacteria genera) selected by the machine learning analysis, considering the category Sampling point.

**Supplementary_Data_S4.csv**

Volatility analysis of Shannon diversity for sampling points over time.
